# The Effects of the Endophytic Bacterium *Pseudomonas fluorescens* Sasm05 and IAA on the Plant Growth and Cadmium Uptake of *Sedum alfredii* Hance

**DOI:** 10.3389/fmicb.2017.02538

**Published:** 2017-12-19

**Authors:** Bao Chen, Sha Luo, Yingjie Wu, Jiayuan Ye, Qiong Wang, Xiaomeng Xu, Fengshan Pan, Kiran Y. Khan, Ying Feng, Xiaoe Yang

**Affiliations:** ^1^MOE Key Laboratory of Environment Remediation and Ecological Health, College of Environmental and Resource Sciences, Zhejiang University, Hangzhou, China; ^2^Zhejiang Bestwa EnviTech Co., Ltd., Post-Doctoral Research Center, Hangzhou, China

**Keywords:** hyperaccumulator, gene expression, metal transporter, plant growth-promoting bacteria (PGPB), Cd

## Abstract

Endophytic bacteria have received attention for their ability to promote plant growth and enhance phytoremediation, which may be attributed to their ability to produce indole-3-acetic acid (IAA). As a signal molecular, IAA plays a key role on the interaction of plant and its endomicrobes. However, the different effects that endophytic bacteria and IAA may have on plant growth and heavy metal uptake is not clear. In this study, the endophytic bacterium *Pseudomonas fluorescens* Sasm05 was isolated from the stem of the zinc (Zn)/cadmium (Cd) hyperaccumulator *Sedum alfredii* Hance. The effects of Sasm05 and exogenous IAA on plant growth, leaf chlorophyll concentration, leaf Mg^2+^-ATPase and Ca^2+^-ATPase activity, cadmium (Cd) uptake and accumulation as well as the expression of metal transporter genes were compared in a hydroponic experiment with 10 μM Cd. The results showed that after treatment with 1 μM IAA, the shoot biomass and chlorophyll concentration increased significantly, but the Cd uptake and accumulation by the plant was not obviously affected. Sasm05 inoculation dramatically increased plant biomass, Cd concentration, shoot chlorophyll concentration and enzyme activities, largely improved the relative expression of the three metal transporter families ZRT/IRT-like protein (ZIP), natural resistance associated macrophage protein (NRAMP) and heavy metal ATPase (HMA). Sasm05 stimulated the expression of the *SaHMAs* (*SaHMA2*, *SaHMA3*, and *SaHMA4*), which enhanced Cd root to shoot translocation, and upregulated *SaZIP*, especially *SaIRT1*, expression to increase Cd uptake. These results showed that although both exogenous IAA and Sasm05 inoculation can improve plant growth and photosynthesis, Sasm05 inoculation has a greater effect on Cd uptake and translocation, indicating that this endophytic bacterium might not only produce IAA to promote plant growth under Cd stress but also directly regulate the expression of putative key Cd uptake and transport genes to enhance Cd accumulation of plant.

## Introduction

Plant growth-promoting bacteria (PGPB) have recently attracted wide attention, because PGPB can effectively increase the plant biomass and the efficiency of heavy metal phytoextraction (Rajkumar et al., 2009; Glick, 2010, 2012, 2014; Bashan et al., 2013; Ma et al., 2016; Santoyo et al., 2016). Many studies have shown that the supplementary effects of PGPB on heavy metal phytoextraction were related to their capacity to promote plant growth, the production of indole-3-acetic acid (IAA), 1-aminocyclopropane-1-carboxylic acid (ACC) deaminase, siderophores, antibiotics and phosphorus (P) solubilization (Penrose and Glick, 2003; Khalid et al., 2004; Hynes et al., 2008; Glick, 2010, 2012, 2014; Bashan et al., 2013). For instance, *Pseudomonas azotoformans* ASS1 could protect plants against abiotic stresses and help plants to thrive in semiarid ecosystems, accelerate the phytoremediation process in metal-polluted soils, and significantly enhance the chlorophyll content and improve the accumulation, bio-concentration factor and biological accumulation coefficient of metals (Ma et al., 2017).

As a phytohormone, IAA is known to be involved in root imitation, cell division and cell enlargement (Teale et al., 2006). It can not only found in plants but also reported to be synthesized in microorganisms (Kazan, 2013). The inoculation of IAA-producing endophytic bacteria has been demonstrated as a promising way to enhance plant biomass, root length, root tip number and root surface area (Chen et al., 2014a; Ali et al., 2017). The regulation of IAA secreted by PGPB and the plant is considered to be an important cause of growth promotion. For example, the bacterial endophyte *Sphingomonas* sp. LK11 isolated from the leaf of *Tephrosia apollinea*, which produces IAA (11.23 ± 0.93 μM mL^-1^) and gibberellins, promoted the growth of tomato (Khan et al., 2014). IAA produced by PGPB might play an extremely important role as a growth regulating substance that drives root hair and cotyledon cell expansion during seedling development (Strader et al., 2010). And even though the concentration of IAA production from various endophytic bacteria are different, IAA synthesis in both plant and microbe were affected by their interaction (Jasim et al., 2014). For instance, an average of 35 μg mL^-1^ IAA was produced in the five endophytic bacteria isolated from *Piper nigrum*, the yield of IAA was drastically increased around 20-fold to 869 μg mL^-1^ which can be due to the induction of the endophytic IAA biosynthetic pathway by the host plant metabolites (Jasim et al., 2014). Additionally, several reports showed that endophytes may also alter plant auxin synthesis (Kazan, 2013). And the cross-border regulation of microbes and their products on the plant IAA signal system is considered as the main mechanism that promotes lateral root development and relieves plant stress (Glick, 2012; Duca et al., 2014). Recent results indicated that IAA-overproducing endophytes had shown many transcriptional changes naturally occurring in nitrogen-fixing root nodule (Defez et al., 2016) and the high expression of *nifH* gene coding for the nitrogenase iron protein, moreover, they could increase nitrogenase activity of rice (Defez et al., 2017). Except for growth promoting, IAA can also affect plant heavy metal uptake and translocation. In Arabidopsis, exogenous auxin could enhance Cd^2+^ fixation in the root cell wall, decrease Cd^2+^ translocation from root to shoot thus to alleviate Cd toxicity (Zhu et al., 2013). Therefore, the IAA producing trait of endophytic bacteria may have multiple consequences in plant–microbe interaction.

Many studies have demonstrated that plants take up Cd primarily by the iron (Fe), zinc (Zn), manganese (Mn), and calcium (Ca) pathways in the roots (Clemens et al., 2013). IRT1 is metal ion transporter with a broad substrate range localized in the plasma membrane in *Arabidopsis thaliana* that can transport Fe, Zn, Mn, Cd, and cobalt (Co) (Korshunova et al., 1999). IRT1 is expressed in the plasma membrane of root epidermal cells (Vert, 2002) and is likely to be involved in Cd uptake. Other metal transporters of the ZRT-IRT-like Protein (ZIP) family (e.g., AtZIP1 and AtZIP2) have been indicated to play a role in Zn and Mn uptake (Milner et al., 2013). In *Arabidopsis thaliana*, P_1B_-type ATPases are known as Heavy Metal ATPase (HMA) and are the major transporters for root-to-shoot Cd translocation (Wong and Cobbett, 2009). In the HMA family, HvHMA2 is a plasma membrane P1B-ATPase from barley that functions in Zn/Cd root-to-shoot transport (Barabasz et al., 2013), AtHMA3 participates in the vacuolar storage of Cd (Morel et al., 2009), and AtHMA4 encodes the export protein responsible for loading Zn and Cd into the xylem vessels, thus controlling root to shoot translocation in Arabidopsis (Wong and Cobbett, 2009). We recently isolated and functionally characterized a tonoplast-localized SaHMA3 gene from *Sedum alfredii* Hance (*S. alfredii*), which had a significantly higher constitutive expression level. SaHMA3 is a Cd-specific transporter with the ability to transport both Cd and Zn (Zhang et al., 2016). Similar results were also obtained in *S. plumbizincicola* (Liu et al., 2017). In addition to ZIP and the HMA family, the Natural Resistance Associated Macrophage Protein (NRAMP) family play an important role in regulating metal ion transport (Sasaki et al., 2012). The plasma membrane-localized OsNramp5 is a major transporter for Cd uptake in rice (Ishimaru et al., 2012; Sasaki et al., 2012). AtNramp3 and AtNramp4 are located in vascular tissues and are both related to the mobilization of vacuolar Cd (Thomine et al., 2003; Lanquar et al., 2010). In Arabidopsis, *Nramp1, 3, 4*, and *6* were shown to confer Cd sensitivity in yeast (Thomine et al., 2000; Cailliatte et al., 2009).

Recent studies showed that PGPB could also regulate host plant gene expression. Defez et al. (2017) showed that the inoculation of IAA-overproducing endophytes could significantly up-regulate nitrogenase activity. *Bacillus altitudinis* WR10 could up-regulate the expression of many genes encoding ferritins, which alleviated iron deficiency in plants (Sun et al., 2017). Our results also showed that the inoculation of the IAA-producing endophytic bacteria SaMR12 can not only increase the leaf chlorophyll content as well as the iron and magnesium uptake but also regulate the expression of the three above mentioned heavy metal transporter genes in *S. alferdii* (Pan et al., 2017). However, few studies were compared the effects of PGPB and exogenous IAA on metal transporter gene expression.

*S. alfredii* is a native of China and a Zn/Cd hyperaccumulator. Based on the data from RNA-seq (Gao et al., 2013), we cloned a series of transporter genes (Zhang et al., 2016), but the mechanism of Cd hyperaccumulation in this plant is still not fully understood. Many endophytic bacteria have been isolated (Zhang et al., 2012) and they might also contribute to Cd hyperaccumulation in plants. In the present study, we investigated (1) the effects of the endophytic bacterium Sasm05 on plant growth and Cd uptake; (2) the effects of IAA and its transport inhibitor naphthylphthalamic acid (NPA) on plant growth and Cd uptake; and (3) the effects of Sasm05, IAA and NPA on the expression of selected transporter genes, to elucidate the functional contribution of IAA to plant–endophytic bacteria interactions.

## Materials and Methods

### Plant Materials and Endophytic Bacterium Sasm05

#### Plant Materials

The plant *S. alfredii* was collected from an old Pb/Zn mined site in Quzhou city, Zhejiang Province of China (Yang et al., 2004), and healthy and uniform shoots were selected and cultured hydroponically. After growth for 12 days in distilled water, the plants were subjected to 4 days exposure to one-half strength Hoagland nutrient solution, continuously aerated and renewed every 3 days. Plants were grown in an environmentally controlled growth chamber with a temperature range of 23–26°C, relative humidity 60% and light intensity of 180 μM m^-2^ s^-1^ during a 14/10 h day/night duration. Single shoot tips were excised and grown as described above for another generation to remove the heavy metals.

#### Isolation and Identification of Sasm05

Healthy plants of *S. alfredii* together with soil were collected from six different points of the mined site, put into a sterile bag and sealed. After come back to the lab, the bacteria were isolated immediately and the other samples were stored at 4°C. The whole plant was washed with tap water for 30 min, and the roots, rotten leaves as well as diseased tissues were removed, and thus only the green healthy parts were preserved. Then the whole process was carried out in a super clean bench. First the plant tissue was washed with distilled water at least three times, 3 min each time. Later the washed tissue was immersed in 75% ethanol to maintain 3 min, sterile water washed 3 times, and then soaked in of 3% NaOCl (Cl^-^ concentration) for 3 min, sterile water washed for five times. The obtained surface sterile tissues were placed on the sterilized filter paper, and the excess water was absorbed. The stems were sliced into thin slices and laid on the solid culture dish containing 20 mL Petri plates of Luria–Bertani’s (LB) medium. The sealed film was used to seal the culture dish and placed in 30°C for dark culture. In order to verify the effectiveness of the *in vitro* sterilization process, 200 μL washed water of the last time was evenly coated on the LB solid medium, and no colony growth treatment was used as effective surface sterilization. The colonies on the plant tissue were picked out with inoculation needle, purified in LB solid medium, and cultured at 30°C for 3 days, then the monoclonal strain was obtained (Supplementary Materials). A single colony was placed on a LB solid medium, 3 replicates per plant, cultured for 48 h and stored at 4°C for further analysis.

After purification and pathogenicity identification, single clone of candidate endophytic bacteria were selected and inoculated to 10 mL liquid LB medium, cultured at 37°C overnight. 1 mL bacteria suspension were put into a 1.5 mL centrifuge tube, 12000 rpm, 3 min. Then the precipitate were collected and washed by sterile water for two times. The genomic DNA was extracted with a rapid bacterial genomic DNA isolation kit (Sangon Biotech, China). Universal primers for bacteria were used for polymerase chain reaction (PCR) amplification; the forward primer was (27f: 5′-AGAGTTTGATCCTGGCTCAG-3′) and the reverse primer was (1492r: 5′-GGTTACCTTGTTACGACTT-3′). The amplified DNA was purified with a DNA purification kit (Sangon Biotech) and sequencing was performed at the Huada biotechnology company (Guangzhou, China). The 16S rDNA sequence was compared with sequences in the GenBank database using the National Center for Biotechnology Information (NCBI) Basic Local Alignment Search Tool - nucleotide (BLASTn) program^1^ (Weisburg et al., 1991). The strain was named Sasm05 and has been preserved in the China General Microbiological Culture Collection Center with preservation number is CGMCC 12173 (Supplementary Materials).

The ability of bacteria to produce siderophore and ACC deaminase as well as heavy metal resistance were investigated according to Sheng et al. (2008). And IAA production and phosphate solubilizing enzyme were determined according to Tiwari et al. (2016).

#### GFP Labeling and Colonization of Sasm05

The green fluorescent protein (GFP) was used to label Sasm05 according to Zhang et al. (2013). Sasm05 was inoculated into 250 mL Erlenmeyer flasks containing 150 mL sterilized LB liquid medium and cultivated aerobically in an orbital rotary shaker (200 rpm) at 30°C for 24 h. The cells were collected by centrifugation, washed three times with 0.85% sterile saline, and resuspended to an OD = 1.0. Uniform plant seedlings were selected, and the roots were immersed in the labeled Sasm05 suspension for 2 h, then transferred to 10 μM Cd-containing Hoagland medium. The roots were imaged by Laser Scanning Confocal Microscopy (LSCM) 6 d after inoculation.

### Experiment Design and Analysis

After 4 weeks of preculture, the roots of uniform young plants were immersed in each treatment for 4 h: (1) Control treatment (sterilized deionized water), (2) Sasm05 (10^7^ CFU /mL), (3) Sasm05 and 10 μM IAA transport inhibitor (NPA), (4) 1 μM IAA, (5) 1 μM IAA and 10 μM NPA, and (6) 10 μM NPA, then exposed to 10 μM Cd Hoagland nutrient solution with (treatments 3, 5, and 6) or without NPA (treatments 1, 2, and 4). 4 plants were transferred into a 2.5 L black plastic bucket as one treatment, and each treatment was repeated six times.

#### Plant Harvest and Weights

The hydroponic plant cultures were harvested after 7 days for gene expression analysis or 30 days for Cd uptake analysis, and the shoots and roots were washed with deionized water. The fresh weights (FW) and the dry weights (DW) of the roots and shoots were recorded before and after oven-drying at 65°C for 48 h.

#### Cd Concentration Determination

The plant roots were soaked in 20 mM Na_2_-EDTA for 15 min to remove the Cd ions adhering to the root surfaces. The shoots and roots were dried and powered as much as possible. A final sample of 0.1 g was digested by adding 5 mL HNO_3_ and 1 mL HClO_4_ in a boiled Polytetrachloroethylene cup at 180°C for 8 h. The digested samples were diluted with deionized water, filtered through a filter membrane (13 mm, 0.22 μm), and the Cd concentration was determined using an Inductively Coupled Plasma Mass Spectrometer (ICP-MS, Agilent 7500a, United States).

#### Chlorophyll Concentration Determination

Acetone and ethyl alcohol were mixed in a ratio of 2:1, fresh leaf (without veins and stalks) samples (0.20 g each) were placed into this mixture (20 mL) in the dark for 24 h before the extracts were measured for the absorbance at wavelengths of 663 and 645 nm using an ultraviolet spectrophotometer (Lambda 350V-vis, PerkinElmer, Singapore) (Chen et al., 2014b). The Chl concentration (g kg^-1^) was calculated using the following equations: Total Chl = [8.02 × A663] + [20.21 × A645].

#### Enzyme Activity Determination

For Ca^2+^-ATPase, 3 mL of total reaction mixture containing 0.3 mL of crude mitochondrial extract, 30 mmol L^-1^ Tris-HCl buffer (pH 8.0), 3 mmol L^-1^ Mg_2_SO_4_, 0.1 mmol L^-1^ Na_3_VO_4_, 50 mmol L^-1^ NaNO_3_, 3 mmol L^-1^ Ca(NO_3_)_2_ and 0.1 mmol L^-1^ ammonium molybdate was employed. The reaction was initiated by the addition of 100 μL of 30 mmol L^-1^ trichloroacetic acid after 20 min of incubation at 37°C. One unit of Ca^2+^-ATPase activity was defined as the release of 1 mol of phosphorus in absorbance per minute at 660 nm under the assay conditions (Jin et al., 2013).

For Mg^2+^-ATPase, a reaction mixture containing 50 mM Tris-HCl, pH 8.8, 33% methanol, 4 mM ATP, 4 mM MgCl_2_ and thylakoids at 30 μg Chl/mL in total volume of 1.0 mL was employed. After incubation at 37°C for 2 min, the reaction was terminated by the addition of 0.1 ml of 20% TCA, then the release of Pi was determined (Ren et al., 1995).

#### Relative Gene Expression Analysis

Shoot and root tissues were collected after 7 days of treatment and immediately frozen in liquid nitrogen prior to total RNA extraction. Total RNA was isolated using TRIzol (Invitrogen). The first-strand cDNA was synthesized with a 10 μL reaction system according to the instructions for the TAKARA PrimeScript RT Reagent Kit (Perfect for Real Time) (TaKaRa Biotechnology, Dalian, China). For a real-time RT-PCR analysis, 1 μL 10-fold-diluted cDNA was used for the quantitative analysis of gene expression performed with SYBR PremixExTaq (Takara), and the pairs of gene-specific primers used were the same as in Pan et al. (2017). The expression data were normalized to the expression of actin (forward: 5′-TGTGCTTTCCCTCTATGCC-3′; reverse: 5′-CGCTCAGCAGTGGTTGTG-3′).

A Mastercycler ep realplex2 Real Time PCR machine (Eppendorf, Hamburg, Germany) with the default program (2 min at 50°C and 10 min at 95°C followed by 40 cycles at 95°C for 30 s, 55°C for 30 s, and 72°C for 30 s.) were employed for quantitative RT-PCR analysis with a reaction mixture volume of 20 μL in an optical 96-well plate. A control was also included in each plate with 2 μL of RNase-free water as a template. Three technical replicates were contained in each plate. Specificity verification of the PCR amplification dissociation and the PCR efficiency curves were determined for each candidate reference gene prior to the quantitative RT-PCR evaluation of these genes in *S. alfredii*. The relative quantification analysis was performed using the comparative ΔΔCt method and the formula was: Fold induction = 2^-ΔΔC_T_^, as described by Winer et al. (1999). To evaluate the gene expression level, the results were normalized using Ct values obtained from actin cDNA amplifications run on the same plate.

### Statistical Analysis

Three replicates were used and analyzed independently for each treatment. The data was analyzed using OriginPro 8, and it was analyzed statistically by a one-way analysis of variance (ANOVA). Significantly different means were indicated by Fisher’s least significant difference (LSD) test and Duncan’s multiple range test at the *P <* 0.05 level.

## Results

### Sasm05 Isolation, *gfp*-Tagging and Its Colonization

Sasm05, an endophytic bacterium isolated from surface sterilized stems of *S. alfredii*, was identified as a gram-negative bacterium and a *Pseudomonas fluorescens* by 16S rRNA array. It could utilize tryptophane for growing and produce IAA (15–50 μg mL^-1^). It also could utilize ACC as the sole nitrogen source and show relatively high levels of ACC deaminase activity (**Table 1**).

**Table 1:** Characteristics of the endophytic bacterium *Pseudomonas fluorescens* Sasm05.

Strain	Data
Bacteria name	Sasm05
Genus affiliation	*Pseudomonas*
Source	Stem
16S rRNA gene accession number	NC_007492.2
Heavy metal resistance (mg L^-1^)	Pb^2.5^, Zn^30^, Cd^2.0^
IAA-production	+
ACCase production	+
Phosphate solubilization	-
Siderophore production	+
Cellulase activity	-
Pectinase activity	-
Antibiotics (μg mL^-1^)	Amp^r(100)^

**FIGURE 1:**
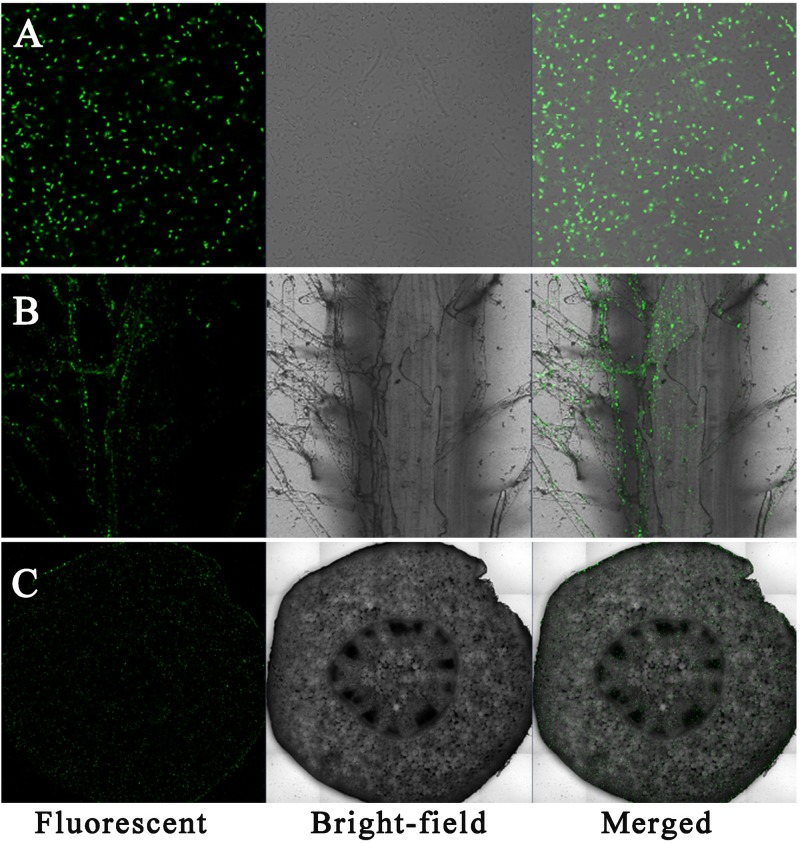
Colonization of *gfp*-tagged Sasm05 in plant. **(A)** *gfp*-tagged Sasm05 strains on root surface, **(B)** colonized in the roots and **(C)** colonized in the stems. LSCM images show bacterial GFP fluorescence in green. Bars = 25 μM.

Sasm05 was successfully tagged by GFP (**Figure 1A**). Laser scanning confocal microscopy (LSCM) showed that under 10 μM Cd treatment, *gfp*-tagged Sasm05 colonized the root surface (**Figure 1B**) and evenly distributed in the plant stem as shown by the cross-section profile of the stem (**Figure 1C**).

### Effect of IAA and Sasm05 on the Plant Growth

**FIGURE 2:**
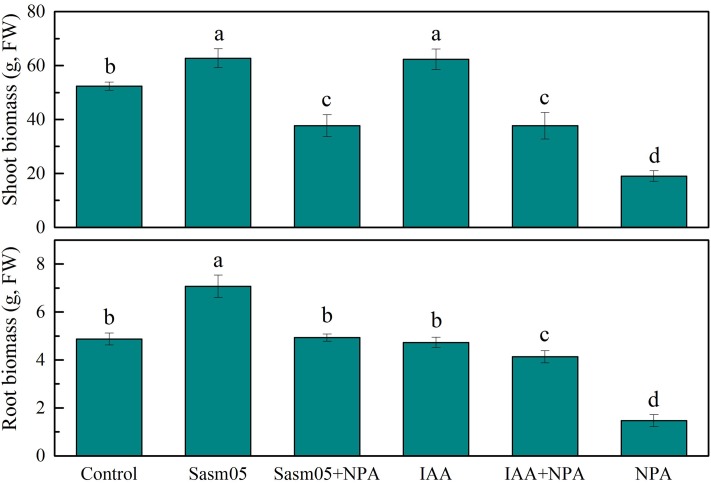
Effects of Sasm05 and IAA on the plant biomass. Error bars represent the standard deviation (SD) of three individual replicates. The different letters on the error bars indicate significant differences among the treatments at *p* < 0.05.

Plants were exposed to 10 μM Cd for 30 days and no visible phytotoxicity of Cd was observed. The shoot and root biomass (expressed as the fresh weight) were significantly (*p <* 0.05) enhanced by 20% and 45% by Sasm05 inoculation (**Figure 2**). IAA treatment could increase the shoot biomass by 19% but had no obvious effect on root biomass (**Figure 2**). However, the plant biomass was significantly inhibited by 64% and 70% (*p* < 0.05) in the 10 μM NPA treatment, and IAA or Sasm05 could alleviate this inhibition to some extent (**Figure 2**).

### Effect of IAA and Sasm05 on Cd Concentration of Plant

**FIGURE 3:**
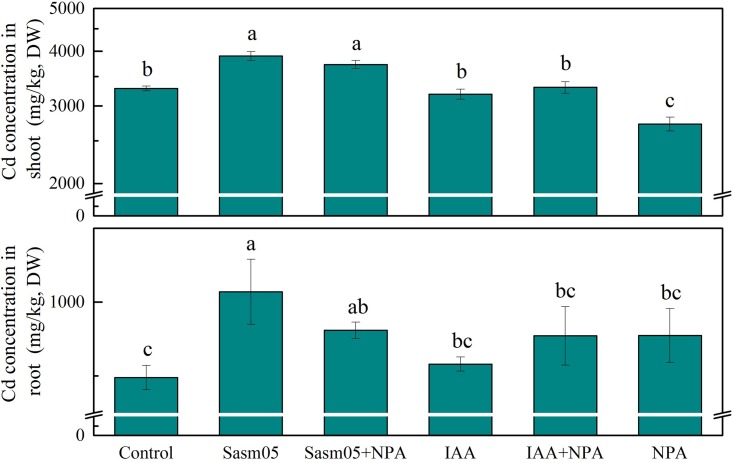
Effects of Sasm05 and IAA on Cd concentration. Error bars represent the standard deviation (SD) from of three individual replicates. The different letters on the error bars indicate significant differences among the treatments at *p* < 0.05.

After inoculation with Sasm05, the Cd concentration in *S. alfredii* was significantly increased by 19% in the shoots and 59% in the roots, and even with an additional NPA treatment, the Cd concentration was still increased by 13% in the shoots and 37% in the roots (**Figure 3**). However, IAA treatment could not increase the Cd concentration compared to the control either whether NPA was added or not. In the NPA treatment, the Cd concentration was significantly decreased by 17% in the shoots and no changes were observed in the roots.

**FIGURE 4:**
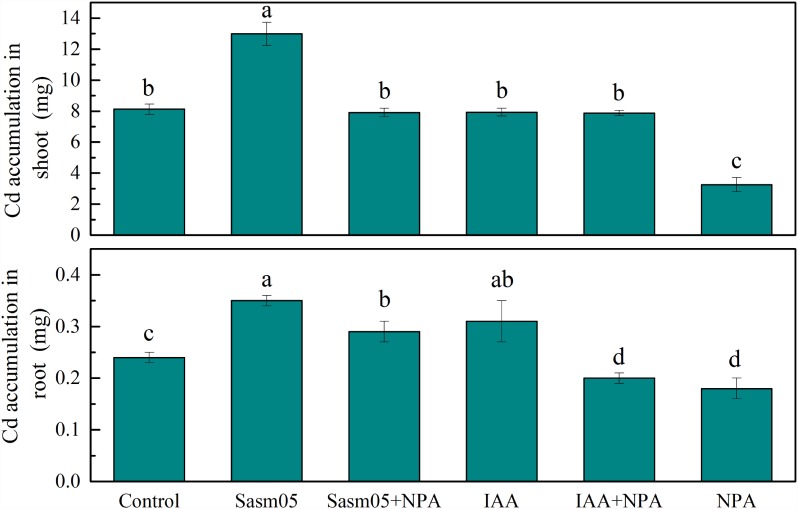
Effects of Sasm05 and IAA on Cd accumulation. Error bars represent the standard deviation (SD) from of three individual replicates. The different letters on the error bars indicate significant differences among the treatments at *p* < 0.05.

Cd accumulation in *S. alfredii* was significantly increased by 60% in the shoots and 46% in the roots with Sasm05 treatment (**Figure 4**). In the shoots, IAA or a combined NPA treatment showed no effect on the Cd accumulation, while it decreased by 60% when treated with NPA alone. In the roots, Sasm05 combined with NPA could increase Cd accumulation by 21%, while IAA combined with NPA or treated with NPA alone decreased the Cd accumulation by 17 and 25%, respectively.

### Effect of IAA and Sasm05 on Leaf Chlorophyll Concentration

**FIGURE 5:**
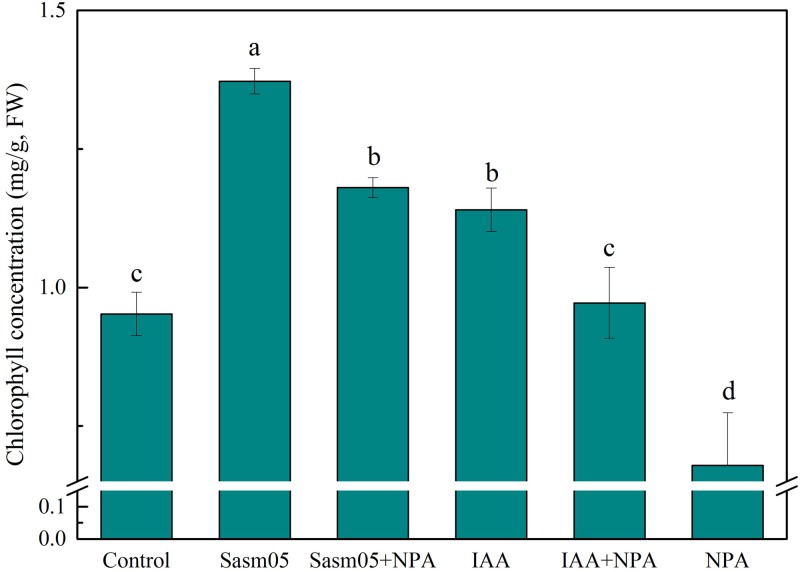
Effects of Sasm05 and IAA on plant chlorophyll concentration. Error bars represent the standard deviation (SD) from of three individual replicates. The different letters on the error bars indicate significant differences among the treatments at *p* < 0.05.

Both Sasm05 and IAA had a positive effect on the content of chlorophyll (*P <* 0.05), which was increased by 24–44 and 20% with Sasm05 and IAA treatments, respectively (**Figure 5**). Combined with NPA treatment, Sasm05 showed a higher chlorophyll concentration (18%) than when combined with an IAA treatment. Treatment with NPA alone resulted in a reduction of the chlorophyll concentration by 40%, indicating that NPA can inhibit the synthesis of chlorophyll (**Figure 5**).

### Effect of IAA and Sasm05 on Leaf Mg^2+^-ATPase and Ca^2+^-ATPase

**FIGURE 6:**
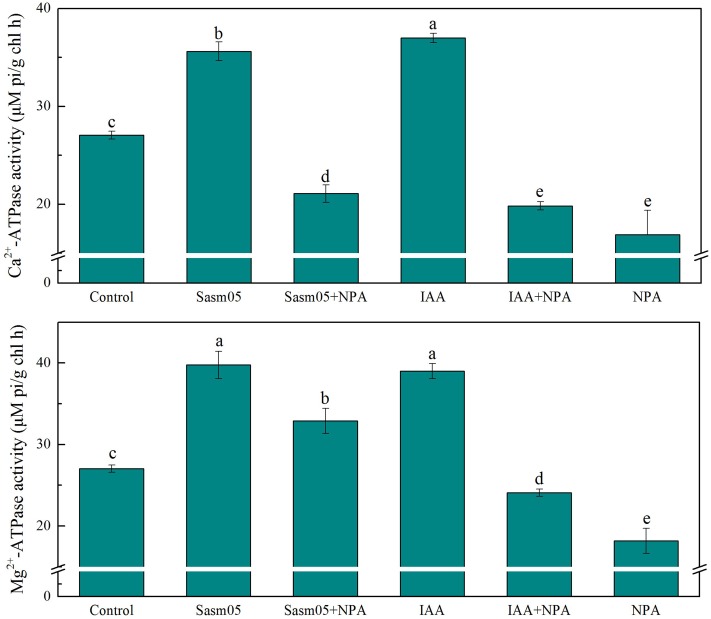
Effects of Sasm05 and IAA on leave Mg^2+^-ATPase activity and Ca^2+^-ATPase activity. Error bars represent the standard deviation (SD) from of three individual replicates. The different letters on the error bars indicate significant differences among the treatments at *p* < 0.05.

ATPases are the main transport proteins and energy source. Ca^2+^-ATPase was often affected by changes in the growth environment. In this experiment, both Sasm05 and IAA treatment could significantly increase the activity of Ca^2+^-ATPase by 32% and 37%, and IAA treatment was better than Sasm05 inoculation. All the NPA treated plants had lower Ca^2+^-ATPase levels than the control, which were decreased by 22% when combined with Sasm05, by 27% when combined with IAA, and by 38% when treated with NPA alone (**Figure 6**).

The Mg^2+^-ATPase activity was significantly enhanced by 47% and 22% in the Sasm05 and Sasm05 + NPA treatments, respectively (**Figure 6**). The biological activity of the Mg^2+^-ATPase was also enhanced by 46% by IAA, but it was significantly decreased by 33% in the IAA + NPA treatment (**Figure 6**).

### The Relative Expression Levels of Metal Transporter Genes

**FIGURE 7:**
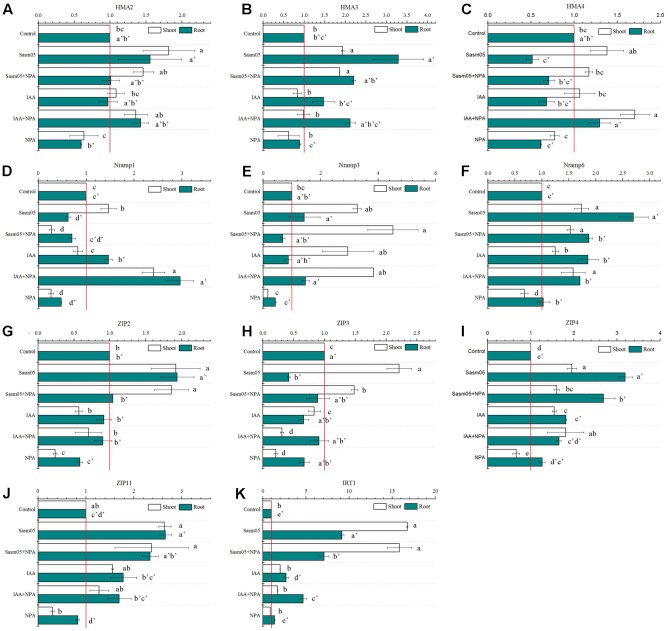
Effects of Sasm05 and IAA on relative expression levels of SaHMAs **(A–C)**, SaNramps **(D–F)** and SaZIPs **(G–K)** genes. Error bars represent the standard deviation (SD) from of three individual replicates. The different letters on the error bars indicate significant differences among the treatments at *p* < 0.05.

The quantitative RT-PCR results revealed that the transcripts of these metal transporter genes were highly induced by Sasm05, and the *IRT1* transcript levels in the Sasm05 treatment in the shoots and the roots were 17-fold and 9-fold higher, respectively (**Figure 7K**). After inoculation with Sasm05, the expression of *SaHMAs* (*SaHMA2*, *SaHMA3* and *SaHMA4*), *SaNramps* (*SaNramp1*, *SaNramp3*, *SaNramp6*), and *SaZIPs* (*SaZIP2*, *SaZIP3*, *SaZIP4* and *SaZIP11*) in the shoots and *SaHMAs* (*SaHMA2* and *SaHMA3*), *SaNramps* (*SaNramp3* and *SaNramp6*), as well as *SaZIPs* (*SaZIP2*, *SaZIP4* and *SaZIP11*) in the roots was significantly increased (**Figures 7A–K**). IAA treatment had no obvious effect on most genes, but it also increased the expression of *SaNramp3*, *SaNramp6* and *SaZIP4* in the shoots and *SaNramps* (*SaNramp1*, *SaNramp6*), *SaZIPs* (*SaZIP4*, *SaZIP11* and *SaIRT1*) in the roots (**Figures 7A–K**). On the contrary, the expression levels of *SaHMAs* (*SaHMA2* and *SaHMA4*), *SaNramps* (*SaNramp1*, *SaNramp3* and *SaNramp6*), *SaZIPs* (*SaZIP3*, *SaZIP4*, and *SaZIP11*) in the shoots and *SaHMAs* (*SaHMA2*, *SaHMA3* and *SaHMA4*), *SaNramps* (*SaNramp1* and *SaNramp3*), *SaZIPs* (*SaZIP2*, *SaZIP11*, and *SaIRT1*) in the roots were reduced by the addition of NPA (**Figures 7A–K**). However, the addition of NPA and IAA increased the expression levels of *SaHMAs* (*SaHMA2* and *SaHMA4*), *SaNramps* (*SaNramp1*, *SaNramp3*, and *SaNramp6*), *SaZIP4* in the shoots and *SaHMAs* (*SaHMA3* and *SaHMA4*), *SaNramps* (*SaNramp1*, *SaNramp3* and *SaNramp6*), *SaZIPs* (*SaZIP4*, *SaZIP11*, *SaIRT1*) in the roots (**Figures 7A–K**).

## Discussion

### The Plant Growth-Promoting Effects of PGPB and IAA

Previous studies showed that endophyte inoculation improved plant biomass and root growth (Zhang et al., 2013; Chen et al., 2014a,b; Cocozza et al., 2014). In the hydroponic experiment, both Sasm05 and IAA could promote plant growth and enhanced shoot biomass. IAA is a regulator known to stimulate both rapid (e.g., increases in cell elongation) and long-term (e.g., cell division and differentiation) responses in plants (Shi et al., 2009). ACC deaminase can lower plant ethylene levels and thus stimulate plant growth (Glick et al., 2007). Furthermore, siderophores are able to bind metals, improving plant growth and enhancing phytoremediation processes (Rajkumar et al., 2010). Therefore, it was reasonable that Sasm05 treatment showed greater improvement in plant growth and root biomass compared to the exogenous IAA treatment.

As a transport inhibitor of IAA, NPA significantly decreased the root IAA concentration while it increased the shoot IAA concentration (Gong et al., 2014). Ljung et al. (2001) conducted an experiment in which 40 mM NPA was added to the medium, demonstrating that NPA decreased the amount of IAA and leaf expansion probably by feedback inhibition of IAA biosynthesis. Root length and lateral root development was significantly inhibited after the application of 10 μM NPA compared with the absence of NPA (Casimiro et al., 2001). Further study revealed that the role of NPA is not only related to auxin transport but also closely related to the content of ethylene; the inhibitory effect of NPA on the growth of plant roots may be related to an increase in the ethylene content (Rashotte et al., 2000; Keller et al., 2004; Ruzicka et al., 2007). Here, the root biomass was significantly reduced by NPA, which was consistent with the discovery of Barkoulas et al. (2008), for it might have a significant inhibitory effect on the plant lateral roots (Gong et al., 2014). NPA treatment prevents the lateral roots from penetrating the hypodermis due to the hardening of hypodermis cell walls and the disturbance of gravitropism (Ogawa et al., 2017).

### Effect of Sasm05 and IAA on Mg^2+^-ATPase and Ca^2+^-ATPase

Indole-3-acetic acid is the most common, naturally occurring plant hormone in the auxin class (Marathe et al., 2017). Apart from IAA produced by plants, some bacteria hosted in plant showed the ability to produce IAA and promote plant growth. The IAA produced by *Pseudomonas fluorescens* HP72 is proposed to act as a stimulator of cell proliferation and elongation and enhance the host uptake of minerals and nutrients from the soil (Suzuki et al., 2003). This promotion effect was outstanding when the plant was grown in a stressed environment, such as stress from water, temperature, salt, pollutants, and so on (Husen et al., 2016; Ma et al., 2016). Thus, growth parameters including chlorophyll synthesis were suppressed under a stressed environment, and the effect of the stress was greater in plants that received no additional IAA treatment than on those treated with exogenous IAA or IAA-producing PGPB. Inoculation with *Pantoea alhagi* sp. nov. could promote chlorophyll production in the leaves compared to non-inoculated control plants under similar water stress conditions (Chen et al., 2017). Consistently, the addition of Sasm05 and IAA could also significantly increase the chlorophyll concentration (**Figure 5**).

Mg^2+^ and Ca^2+^ are essential to the integrity of the cellular membrane and the intracellular adhesives, and the function of the Ca^2+^-ATPase and Mg^2+^-ATPase are important in the maintenance of the intracellular calcium and magnesium level (de la Torre et al., 2000; Lin et al., 2016). In the present study, the activity of Ca^2+^-ATPase and Mg^2+^-ATPase were measured, and it was found that both Sasm05 and IAA can activate Ca^2+^-ATPase and Mg^2+^-ATPase (**Figure 6**), indicating that both of them can stimulate photosynthesis. The IAA treatment had a growth-stimulating effect via the simulation of the electrogenic activity of the plasmalemma membrane potential H^+^-ATPase and the hormonal effects were mediated by a transient elevation in the level of free Ca^2+^ in the cytosol and generation of reactive oxygen species (Kovaleva et al., 2016). However, all NPA treatments sharply decreased the activity of Ca^2+^-ATPase and Mg^2+^-ATPase, as well as decreasing the leaf chlorophyll content (**Figure 5**), which illustrated that IAA and its transport had dramatic effects on the improvement of plant photosynthesis.

### The Effects of Sasm05 and IAA on Cd Uptake and Transport

Although a lot of literatures indicated that PGPB can increase heavy metal uptake in plant, the opposite results were also not unusual. Mesa et al. (2015) found that endophytic cultivable bacteria of the metal bioaccumulator *Spartina maritima* improve plant growth but not metal uptake in polluted marshes soils. Here we found that Sasm05 increase Cd concentration in both root and shoot while exogenous have no significant effects (**Figure 3**). Therefore, we assumed that it should be related with transporter gene expression regulation.

Many metal transporters and homologous genes involved in metal transport in plants have been identified by genetic and molecular techniques, such as sequence comparison. A suite of metal-sensing regulatory proteins from bacterial sources orchestrated metal homeostasis by allosterically coupling the selective binding of target metals to the activity of the DNA-binding domains for detecting and responding to toxic levels of heavy metals (Furukawa et al., 2015). Although our previous data showed that the IAA-producing PGPB *Sphingomonas* SaMR12 could affect the gene expression of transporters (Pan et al., 2017), the effects of exogenous IAA and the auxin transport inhibitor were the first time compared. In Arabidopsis, AtHMA2 and AtHMA4 are essential for Zn and Cd translocation from the roots to the shoots (Eren, 2004; Verret et al., 2004). AtHMA3 is another heavy metal ATPase transporter that is located in the tonoplast and is associated with Cd and Zn tolerance (Morel et al., 2009). And in *Cucumber*, *CsHMA3*, *CsHMA4* were predominantly expressed in the roots and up-regulated by excess Zn and Cd (Migocka et al., 2015). SaHMA3 of *S. alfredii* was a Cd transporter, constitutively expressed in both shoots and roots, and encoded tonoplast-localized proteins (Zhang et al., 2016). Although no significantly induced expression of *SaHMA2*, *SaHMA3* and *SaHMA4* was observed after addition of IAA or NPA, Sasm05 inoculation increased their expression levels except for *SaHMA4* in the roots (**Figures 7A–C**), indicating that Sasm05 might enhance Cd root to shoot translocation by regulation these genes.

In Arabidopsis, six *AtNramp1-6* genes encode the NRAMP proteins. AtNramp1 played an important role on plant iron homoeostasis (Curie et al., 2000). AtNramp3 and AtNramp4 are localized to the vacuolar membrane and encode tonoplastic proteins with redundant functions (Lanquar et al., 2005). They are metal transporters with a broad range of substrate specificities including Fe, Cd, and Zn (Lanquar et al., 2005, 2010). AtNramp6 is an intracellular Cd transporter that functions inside the cell either by mobilizing Cd from its storage compartment or by taking up Cd into a cellular compartment where it becomes toxic (Cailliatte et al., 2009). However, in rice, OsNramp5 has been reported to be a major transporter for Cd uptake (Ishimaru et al., 2012; Sasaki et al., 2012), and the expression of *OsNramp5* was increased in the roots and shoots in the presence of Cd (Ishimaru et al., 2012). In this research, we found the variation of the transcription levels of *SaNramp1*, *SaNramp3*, and *SaNramp6* in the different treatments are not uniform (**Figures 7D–F**), indicating the function of these genes may also differ. NPA greatly inhibited the expression of these genes, while Sasm05 and IAA increased their expression. Those results indicated that Sasm05 and IAA might help the plant alleviate Cd toxicity to the cells, but more data about the functions of these genes in *S. alfredii* are needed.

Present studies have indicated that the ZIP family is the main membrane transporter responsible for possible Cd^2+^ uptake and transport (Zhu et al., 2013). As expected, the expression levels of ZIP family genes in the shoots and roots were all up-regulated by the Sasm05 treatment, except for *ZIP3* in the roots (**Figures 7G–K**), suggesting that Sasm05 causes more Cd^2+^ to enter the cell. Although the relative expression levels of other genes varied limited in fivefold, the expression of *IRT1* was dramatically induced by Sasm05 by 17-fold in the shoots and 9-fold in the roots (**Figure 7K**), suggesting that Sasm05 promotes plant Cd uptake and transport through up-regulating the expression of the ZIP genes, especially *IRT1*.

## Conclusion

In conclusion, with exposure to Cd, both the endophytic bacterium Sasm05 and exogenous IAA can promote the growth and photosynthesis of *S. alfredii*, and Sasm05 has greater effects on the enhancement of phytoextraction compare with IAA treatment. Moreover, Sasm05 can upregulate the expression of key transporters for Cd uptake, root to shoot translocation as well as detoxification, although IAA can also improve the expression of some transporter genes involved metal uptake and detoxification. These results indicated that the beneficial plant-endophytic bacterial interaction of Sasm05 is but not limited to IAA production. This study analyzed for the first time the effects of PGPB Sasm05 and exogenous IAA on phytoremediation of Cd contaminated soil, through which not only cleared the improvement from IAA treatment, but also illustrated related mechanisms of phytoremediation enhancement from Sasm05. These results will guide our subsequent study on plant growth promoting endophytic bacteria and their application for realizing efficient phytoremediation.

## Author Contributions

All authors listed have made a substantial, direct and intellectual contribution to the work, and approved it for publication. In this research, YF, XY, and BC designed the experiment; BC, SL, YW, FP, QW, and JY performed the experiment; SL, BC, and QW analyzed the data and drafted the manuscript; YF, BC, XX, FP, and KK revised the manuscript.

## Conflict of Interest Statement

The authors declare that the research was conducted in the absence of any commercial or financial relationships that could be construed as a potential conflict of interest.

## Footnotes

1 http://blast.ncbi.nlm.nih.gov
