# Supplementary material for: The Effects of the Endophytic Bacterium Pseudomonas fluorescens Sasm05 and IAA on the Plant Growth and Cadmium Uptake of Sedum alfredii Hance
Source: Front Microbiol. 2017 Dec 19;8:2538. doi: 10.3389/fmicb.2017.02538 (PMC5742199; doi:10.3389/fmicb.2017.02538)
Supplement: Supplementary file 1 [file Image_1.PDF]

Screening process of endophytic bacterium Sasm05

Chen Bao

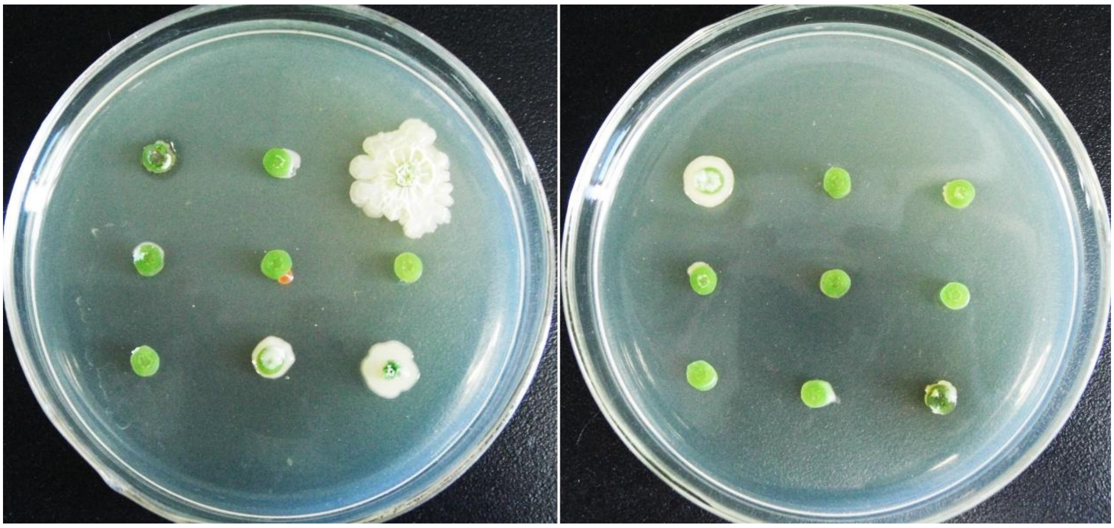

Endophytic bacteria screening on LB nutrient medium

Healthy plants of *S. alfredii* together with soil were collected from six different points of the mined site, put into a sterile bag and sealed. After come back to the lab, the bacteria were isolated immediately and the other samples were stored at 4℃. The whole plant was washed with tap water for 30 min, and the roots, rotten leaves as well as diseased tissues were removed, and thus only the green healthy parts were preserved. Then the whole process was carried out in a super clean bench. First the plant tissue was washed with distilled water at least 3 times, 3 min each time. Later the washed tissue was immersed in 75% ethanol to maintain 3 min, sterile water washed 3 times, and then soaked in of 3% NaOCl (Cl- concentration) for 3 min, sterile water washed for 5 times. The obtained surface sterile tissues were placed on the sterilized filter paper, and the excess water was absorbed. The stems were sliced into thin slices and laid on the solid culture dish containing 20 mL Petri plates of Luria–Bertani's (LB) medium. The sealed film was used to seal the culture dish and placed in 30℃ for dark culture. In order to verify the effectiveness of the in vitro sterilization process, 200 μL washed water of the last time was evenly coated on the LB solid medium, and no colony growth treatment was used as effective surface sterilization. The colonies on the plant tissue were picked out with inoculation needle, purified in LB solid medium, and cultured at 30℃ for 3 d, then the monoclonal strain was obtained. A single colony was placed on

a LB solid medium, 3 replicates per plant, cultured for 48 h and stored at 4 ℃ for further analysis.

Sasm05 Preservation Certificate

The plant growth promoting endophytic bacterium Sasm05 was preserved in CGMCC.

中国微生物菌种保藏管理委员会普通微生物中心
China General Microbiological Culture Collection Center (CGMCC)

地址：北京市朝阳区北辰西路1号院3号　中国科学院微生物研究所　邮政编码：100101
电话：010-64807355　传真：010-64807288　电子邮件：cgmcc@im.ac.cn　http://www.cgmcc.net

受理通知书（收据）
存活性报告书　　用于专利程序的生物材料保存

发出日期 2016 年 03 月 03 日

（请求保藏人或代理人的姓名、地址）

杨肖娥
浙江大学
浙江省杭州市浙江大学紫金港校区农生环B座515

本保藏中心登记入册编号
CGMCC No. 12173

申请专利的发明名称

申请号NO.
申请日期　年　月　日

你（们）提供的请求保藏并注明以下鉴定

参据的生物材料(株)：SM05

上述请求保藏的生物材料（株）附有

☐ 科学描述
☑ 建议的分类命名：荧光假单胞菌
Pseudomonas fluorescens

该生物材料（株）已于 2016 年 03 月 03 日由本保藏中心收到，并登记入册。
根据你（们）的请求，由该日起保存三十年，在期满前收到提供生物材料样品的请求后再延续保存五年。
该生物材料（株）的存活性经本保藏中心于 2016 年 03 月 03 日检测，结果是：

（1）存活 √　（2）失活 /

中国微生物菌种保藏管理委员会普通微生物中心负责人签字和日期

姓名 [signature] 2016 年 04 月 21 日

（印章：中国微生物菌种保藏管理委员会普通微生物中心）

Blast Analysis of Sasm05 V3 Conserved Region

Result:

The V3 conserved region of endophytic bacteria Sasm05 was amplified with primers 27f-1492r, the sequence was most similar with *Pseudomonas fluorescens* Pf0-1(access No. NC 007492.2) compare to the sequence existed in NCBI.

Score = 2556 bits (1384), Expect = 0.0
Identities = 1401/1409 (99%), Gaps = 3/1409 (0%)
Strand=Plus/Minus

Sequence:

GCATATCACCGTGGTACCGTCCTCCGAAGGTTAGACTAGCTACTTCTGGTGCAACCCAC
TCCCATGGTGTGACGGGCGGTGTGTACAAGGCCCGGGAACGTATTCACCGCGACATTC
TGATTCGCGATTACTAGCGATTCCGACTTCACGCAGTCGAGTTGCAGACTGCGATCCGG
ACTACGATCGGTTTTGTGGGATTAGCTCCACCTCGCGGCTTGGCAACCCTCTGTACCGA
CCATTGTAGCACGTGTGTAGCCCAGGCCGTAAGGGCCATGATGACTTGACGTCATCCCC
ACCTTCCTCCGGTTTGTCACCGGCAGTCTCCTTAGAGTGCCCACCATAACGTGCTGGTA
ACTAAGGACAAGGGTTGCGCTCGTTACGGGACTTAACCCAACATCTCACGACACGAGC
TGACGACAGCCATGCAGCACCTGTCTCAATGYTCCCGAAGGCACCAATCCATCTCTGG
AAAGTTCATTGGATGTCAAGGCCTGGTAAGGTTCTTCGCGTTGCTTCGAATTAAACCAC
ATGCTCCACCGCTTGTGCGGGCCCCCGTCAATTCATTTGAGTTTTAACCTTGCGGCCGT
ACTCCCCAGGCGGTCAACTTAATGCGTTAGCTGCGCCACTAAGAGCTCAAGGCTCCCA
ACGGCTAGTTGACATCGTTTACGGCGTGGACTACCAGGGTATCTAATCCTGTTTGCTCC
CCACGCTTTCGCACCTCAGTGTCAGTATCAGTCCAGGTGGTCGCCTTCGCCACTGGTGT
TCCTTCCTATATCTACGCATTTCACCGCTACACAGGAAATTCCACCACCCTCTACCATAC
TCTAGCTTGCCAGTTTTGGATGCAGTTCCCAGGTTGAGCCCGGGGATTTCACATCCAAC
TTAACAAACCACCTACGCGCGCTTTACGCCCAGTAATTCCGATTAACGCTTGCACCCTC
TGTATTACCGCGGCTGCTGGCACAGAGTTAGCCGGTGCTTATTCTGTCGGTAACGTCAA
AATTGCAGAGTATTAATCTACAACCCTTCCTCCCAACTTAAAGTGCTTTACAATCCGAA
GACCTTCTTCACACACGCGGCATGGCTGGATCAGGCTTTCGCCCATTGTCCAATATTCC
CCACTGCTGCCTCCCGTAGGAGTCTGGACCGTGTCTCAGTTCCAGTGTGACTGATCATC
CTCTCAGACCAGTTACGGATCGTCGCCTTGGTGAGCCATTACCTCACCAACTAGCTAAT
CCGACCTAGGCTCATCTGATAGCGCAAGGCCCGAAGGTCCCCTGCTTTCTCCCGTAGG
ACGTATGCGGTATTAGCGTTCCTTTCGAAACGTTGTCCCCCACTACCAGGCAGATTCCT
AGGCATTACTCACCCGTCCGCCGCTGAATCCAGGAGCAAGCTCCTTCATCCGCTCGAC
TGGACGGTCTCTACGACTGCAGGAGCCCC
